# BRCA Germline Mutations in Prostate Cancer: The Future Is Tailored

**DOI:** 10.3390/diagnostics11050908

**Published:** 2021-05-19

**Authors:** Felice Crocetto, Biagio Barone, Vincenzo Francesco Caputo, Matteo Fontana, Ottavio de Cobelli, Matteo Ferro

**Affiliations:** 1 Department of Neurosciences, Reproductive Sciences and Odontostomatology, University of Naples “Federico II”, 80131 Naples, Italy; felice.crocetto@unina.it (F.C.); biagio.barone@unina.it (B.B.); vincitor@me.com (V.F.C.); 2 Division of Urology, European Institute of Oncology, IRCSS, Milan, Via Ripamonti 435, 20141 Milan, Italy; matteo.fontana@ieo.it (M.F.); ottavio.decobelli@ieo.it (O.d.C.); 3 Department of Oncology and Hematology-Oncology, University of Milan, 20122 Milan, Italy

Prostate cancer (PCa) is the second most common neoplasm in men and the fifth leading cause of death worldwide [1]. Despite recent advancements in the diagnosis and treatment of this disease, the incidence of PCa is rising and the clinical outcomes of patients with metastatic castration-resistant prostate cancer (mCRPC) are still poor [2,3]. Excluding advanced age and African American ancestries, the only currently identified risk factor for the development of PCa is a positive family history. A substantial inherited component has been estimated to be present in 40–50% of PCA, and several genetic mutations have been implicated [4]. Among these, BRCA1 and BRCA2 mutations stand out for their renowned role in carcinogenesis in ovarian and female breast cancer, with recent evidence reporting mutated BRCA genes as a risk factor also for the development of male breast cancer [5]. BRCA genes are indeed tumour suppressor factors involved in DNA repair, in particular in the homologous recombination repair (HRR) process of double-strand breaks.

The ability of a cell to maintain genome stability is pivotal to control tumorigenesis. Somatic mutations occur as a result of errors in DNA repair mechanisms or as a direct response to stress, and normally occur during the ageing process: despite being involved in the carcinogenesis of sporadic cancers, these mutations are not passed down to the offspring through gametes, and consequently are not responsible for cancer familiarity. Conversely, a germline mutation involves gametes by definition and is therefore passed down to the offspring. Consequently, the presence of an inherited alteration in oncosuppressors could increase the risk of developing hereditary cancer. The presence of these mutations and their proper identification, both before and after the development of cancer, is relevant to the prevention, diagnosis and even treatment of affected patients [6].

BRCA1-2 germline mutations affect the susceptibility of individuals to carcinogenesis through the two-hit hypothesis: the first hit owing to the inherited pathogenic mutation, and the second hit owing to the somatic inactivation of the second wild-type allele of the oncosuppressor gene. The failure of the BRCA HRR mechanism is due to the combination of germline and somatic mutations that favour the activation of alternative and less effective DNA repair pathways such as base excision repair, nucleotide excision repair and mismatch repair [7]. In addition to their role in preserving genome integrity, BRCA1 has been identified as a coregulator of androgen receptor (AR) in prostatic tissue, which mediates an essential signalling pathway in the development and progression of PCa, while BRCA2 has been identified as a regulator and limiter of the PCa metastatic potential through the downregulation of MMP9 and the inhibition of PI3-kinase/AKT and MAP/ERK pathways [8].

The Cancer Genome Atlas Research Network identified several germline mutations of DNA damage repair (DDR) genes in 333 patients with primary PCa, comprehending BRCA2 (mutated in 13% of cases), ATM (7.3%), MSH2 (2%), and BRCA1 (0.3%) [9]. Although initially the prevalence of BRCA1-2 mutations in PCa has been estimated to be lower than 15%, a recent study by Nicolosi et al. described a prevalence of 24.3% for BRCA2 and 6.4% for BRCA germline mutations in 620 patients with PCa [10]. To further outline the role of these mutations in PCa, it has been estimated that 90% of mCRPC patients present up to 20–25% of mutation in DDR genes pathways [11].

Recognizing the importance of BRCA germline mutations in PCa is particularly relevant in the clinical setting for several reasons, mainly because carriers present an increased lifetime risk of developing a PCa compared to non-carriers, and because BRCA-related PCa is characterized by worse clinical outcomes. Furthermore, BRCA gene alterations have recently been evaluated as predictors of response to both poly ADP-ribose polymerase (PARP) inhibitors and platinum chemotherapy in the metastatic setting. Carrying a BRCA1 mutation is associated with a PCa standardised incidence ratio of 2.35 in comparison to the overall population, while carrying a BRCA2 mutation increases this ratio to 4.45. Similarly, the standardised mortality ratio is increased by a 1.75 and 3.85 factor for BRCA1 and BRCA2 mutation carriers, respectively, in comparison to non-carriers [12]. Another study showed that the estimated lifetime risk of PCa is as high as 20% in BRCA2 and 9.5% in BRCA1 mutation carriers [13]. Moreover, BRCA germline mutations have been associated with more aggressive disease and poorer clinical outcomes, reporting higher Gleason score, nodal involvement and metastatic disease at diagnosis. Cancer-specific survival (CSS) and metastasis-free survival (MFS) at 5 years are also negatively affected, with a 8.6 years CSS in BRCA mutation carriers compared to 15.7 years in non-carriers and a 77% MFS versus 93% [14].

In addition to disease incidence and survival outcomes, the presence of BRCA germline mutations affects the effectiveness of treatments. BRCA2 carriers are indeed more likely to present a tumour requiring active treatment: however, despite radical surgery, 5- and 10-years MFS was, respectively, 89% and 67% for patients withBRCA1 and 2 germline mutations, compared to 97% and 91% for those with the wild-type alleles. Clinical outcomes appear to be even worse when radiotherapy was chosen as the primary treatment, with 57% and 39% of BRCA1-2 carriers free from metastasis at 5 and 10 years compared to the 91% and 80% of non-carriers [15]. Moreover, the PROREPAIR-B study has shown that patients with metastatic PCa and germline BRCA2 mutation become resistant to androgen-deprivation therapy faster than non-carriers (13.2 months versus 28 months), with a halved CSS [16].

Due to these premises, specific treatment strategies are required in order to improve clinical outcomes for BRCA germline mutations carriers. Platinum-based therapy has long been a cornerstone in the treatment of BRCA positive metastatic tumours due to the intrinsic susceptibility of tumour cells to DNA damaging agents. In standardized protocols for PCa, platinum-based chemotherapy has been proposed only for neuroendocrine differentiation, even though different studies have reported an increased sensitivity of BRCA mutated PCa to this agent. Indeed, 75% of BRCA2 carriers have reported a PSA decline > 50% within 12 weeks of treatment initiation compared to 17% of non-carriers, with overall prolonged survival in the first cohort of 18.9 months versus 9.5 months [17]. Similarly, as reported by Schimd et al. 63.9% of patients with BRCA2 mutations reported a PSA decline > 50% compared to 20–30% of non-carriers [18].

Another effective treatment strategy in the BRCA-mutated population comprehends the inhibition of the PARP enzyme complex involved in DNA repair, thus exploiting the deficiency of HRR mechanisms in these tumours to disrupt DNA integrity and cause cell death. Current PARP inhibitors that have been approved for the treatment of mCRPC with BRCA germline mutations are Olaparib and Rucaparib; however, other PARP inhibitors, as Niraparib and Talazoparib, are currently being investigated in this setting. The TRITON2 phase II trial, which evaluated the efficacy of Rucaparib in a population with DDR defects, reported PSA and radiographic responses in 48% and 45%, respectively, in BRCA2 carriers [19]. Similarly, the PROFOUND phase III trial assessed the response to Olaparib compared to androgen receptor signalling inhibitors (ARSi) in mCRPC patients with BRCA1-2 germline mutations, reporting a median progression-free survival of 7.4 months versus 3.55 months [11]. The GALAHAD trial investigated the efficacy of Niraparib in the same setting, reporting a higher objective response rate (ORR) (41% versus 9%) and longer progression-free survival (8.2 months versus 5.3 months) in mCRPC patients with DDR defects receiving ARSi and taxane-based chemotherapy [20]. Finally, the TALAPRO-1 phase II trial (NCT03148795) is currently evaluating Talazoparib in mCRPC patients, reporting an initial ORR of 43.9% for BRCA1-2 mutations carriers compared to 11.8% for ATM mutations carriers.

DDR defects are present in prostate cancer at a higher prevalence than previously recognized. In particular, BRCA germline mutations have implications not only when assessing the risk of developing PCa, but also in the prognosis and the management of this disease. Most of the time, DDR mutation carriers lack a personal or family history of cancers that could be suggestive of an inheritable predisposition. Understanding the real prevalence of BRCA germline mutations could substantially change clinical practice and help to improve therapies and diagnostic protocols in a tailored therapy setting. Routing genetic testing and molecular characterization of patients with either mCRPC or locally advanced PCa could therefore be a feasible approach to select patients who are more likely to respond to targeted agents, minimizing toxicities from unnecessary therapies and tailoring a more efficient patient-based treatment. A multidisciplinary approach should also be considered in these patients to guarantee proper genetic counselling to the carriers. Although the approval of PARP inhibitors in mCRPC is the first step towards a targeted therapy in this subpopulation, further efforts are required to properly evaluate and exploit DDR mutations in PCa.

## References

[1] McNevin C.S., Baird A.-M., McDermott R., Finn S.P. (2021). Diagnostic Strategies for Treatment Selection in Advanced Prostate Cancer. Diagnostics.

[2] Ferro M., Lucarelli G., Crocetto F., Dolce P., Verde A., La Civita E., Zappavigna S., de Cobelli O., Di Lorenzo G., Facchini B.A. (2021). First-line systemic therapy for metastatic castration-sensitive prostate cancer: An updated systematic review with novel findings. Crit. Rev. Oncol..

[3] Buonerba C., Matteo M., Dolce P., Felice C., Verde A., Giuseppe L., Luca S., Sergio F., Angelo V., Alfredo M. (2020). Predictors of efficacy of androgen-receptor-axis-targeted therapies in patients with metastatic castration-sensitive prostate cancer: A systematic review and meta-analysis. Crit. Rev. Oncol..

[4] Angeles A.K., Bauer S., Ratz L., Klauck S.M., Sültmann H. (2018). Genome-Based Classification and Therapy of Prostate Cancer. Diagnostics.

[5] Tedaldi G., Tebaldi M., Zampiga V., Cangini I., Pirini F., Ferracci E., Danesi R., Arcangeli V., Ravegnani M., Martinelli G. (2020). Male Breast Cancer: Results of the Application of Multigene Panel Testing to an Italian Cohort of Patients. Diagnostics.

[6] Pujol P.T., De La Motte Rouge F. (2019). Penault-Llorca, From Targeting Somatic Mutations to Finding Inherited Cancer Predispositions: The Other Side of the Coin. Diagnostics.

[7] Roy R., Chun J., Powell S.N. (2012). BRCA1 and BRCA2: Different roles in a common pathway of genome protection. Nat. Rev. Cancer.

[8] Moro L., Arbini A.A., Yao J.L., Di Sant’Agnese P.A., Marra E., Greco M. (2008). Loss of BRCA2 promotes prostate cancer cell invasion through up-regulation of matrix metalloproteinase-9. Cancer Sci..

[9] Cancer Genome Atlas Research Network (2015). The Molecular Taxonomy of Primary Prostate Cancer. Cell.

[10] Nicolosi P., Ledet E., Yang S. (2019). Prevalence of Germline Variants in Prostate Cancer and Implications for Current Genetic Testing Guidelines. JAMA Oncol..

[11] De Bono J., Mateo J., Fizazi K., Saad F. (2020). Olaparib for Metastatic Castration-Resistant Prostate Cancer. N. Engl. J. Med..

[12] Nyberg T., Frost D., Barrowdale D., Evans D.G., Bancroft E., Adlard J., Ahmed M., Barwell J., Brady A.F., Brewer C. (2020). Prostate Cancer Risks for Male BRCA1 and BRCA2 Mutation Carriers: A Prospective Cohort Study. Eur. Urol..

[13] Agalliu I., Gern R., Leanza S., Burk R.D. (2009). Associations of High-Grade Prostate Cancer with BRCA1 and BRCA2 Founder Mutations. Clin. Cancer Res..

[14] Castro E., Goh C., Olmos D., Saunders E., Leongamornlert D., Tymrakiewicz M., Mahmud N., Dadaev T., Govindasami K., Guy M. (2013). Germline BRCA Mutations Are Associated with Higher Risk of Nodal Involvement, Distant Metastasis, and Poor Survival Outcomes in Prostate Cancer. J. Clin. Oncol..

[15] Castro E., Goh C., Leongamornlert D., Saunders E., Tymrakiewicz M., Dadaev T., Govindasami K., Guy M., Ellis S., Frost D. (2015). Effect of BRCA Mutations on Metastatic Relapse and Cause-specific Survival After Radical Treatment for Localised Prostate Cancer. Eur. Urol..

[16] Castro E., Romero-Laorden N., del Pozo A. (2019). PROREPAIR-B: A Prospective Cohort Study of the Impact of Germline DNA Repair Mutations on the Outcomes of Patients with Metastatic Castration-Resistant Prostate Cancer. J. Clin. Oncol..

[17] Pomerantz M.M., Spisák S., Jia L., Cronin A.M., Csabai I., Ledet E., Sartor A.O., Rainville I., Ba E.P.O., Herbert Z.T. (2017). The association between germline BRCA2 variants and sensitivity to platinum-based chemotherapy among men with metastatic prostate cancer. Cancer.

[18] Schmid S., Omlin A., Higano C., Sweeney C., Chanza N.M., Mehra N., Kuppen M.C.P., Beltran H., Conteduca V., De Almeida D.V.P. (2020). Activity of Platinum-Based Chemotherapy in Patients with Advanced Prostate Cancer With and Without DNA Repair Gene Aberrations. JAMA Netw. Open.

[19] Abida W., Patnaik A., Campbell D., Shapiro J., Bryce A.H., McDermott R., Sautois B., Vogelzang N.J., Bambury R.M., Voog E. (2020). Rucaparib in Men with Metastatic Castration-Resistant Prostate Cancer Harboring a BRCA1 or BRCA2 Gene Alteration. J. Clin. Oncol..

[20] Smith M., Sandhu S., Kelly W., Scher H., Efstathiou E., Lara P., Yu E., George D., Chi K., Saad F. (2019). Pre-specified interim analysis of GALAHAD: A phase II study of niraparib in patients (pts) with metastatic castration-resistant prostate cancer (mCRPC) and biallelic DNA-repair gene defects (DRD). Ann. Oncol..

